# Changes in Timing and kinematics of goal directed eye-hand movements in early-stage Parkinson’s disease

**DOI:** 10.1186/2047-9158-2-1

**Published:** 2013-01-09

**Authors:** Danya Muilwijk, Simone Verheij, Johan JM Pel, Agnita JW Boon, Johannes van der Steen

**Affiliations:** 1 Department of Neuroscience, Erasmus MC, PO Box 2040,, 3000, CA, Rotterdam, The Netherlands; 2 Department of Neurology, Erasmus MC, PO Box 2040, 3000, CA, Rotterdam, The Netherlands

## Abstract

**Objective:**

Many daily activities involve intrinsic or extrinsic goal-directed eye and hand movements. An extensive visuomotor coordination network including nigro-striatal pathways is required for efficient timing and positioning of eyes and hands. The aim of this study was to investigate how Parkinson’s disease (PD) affects eye-hand coordination in tasks with different cognitive complexity.

**Methods:**

We used a touch screen, an eye-tracking device and a motion capturing system to quantify changes in eye-hand coordination in early-stage PD patients (H&Y < 2.5) and age-matched controls. Timing and kinematics of eye and hand were quantified in four eye-hand coordination tasks (pro-tapping, dual planning, anti-tapping and spatial memory task).

**Results:**

In the pro-tapping task, saccade initiation towards extrinsic goals was not impaired. However, in the dual planning and anti-tapping task initiation of saccades towards intrinsic goals was faster in PD patients. Hand movements were differently affected: initiation of the hand movement was only delayed in the pro-tapping and dual planning task. Overall, hand movements in PD patients were slower executed compared to controls.

**Interpretation:**

Whereas initiation of saccades in an extrinsic goal-directed task (pro-tapping task) is not affected, early stage PD patients have difficulty in suppressing reflexive saccades towards extrinsic goals in tasks where the endpoint is an intrinsic goal (e.g. dual planning and anti-tapping task). This is specific for eye movements, as hand movements have delayed responses in the pro-tapping and dual planning task. This suggests that reported impairment of the dorsolateral prefrontal cortex in early-stage PD patients affects only inhibition of eye movements. We conclude that timing and kinematics of eye and hand movements in visuomotor tasks are affected in PD patients. This result may have clinical significance by providing a behavioral marker for the early diagnosis of PD.

## Introduction

In daily life, even simple visually guided motor behavior such as pressing a button, requires a well-functioning network of many subcortical and cortical regions, including the nigro-striatal pathways. These areas form an integrated network that allows very precise visuomotor coordination of eye and hand movements [1]. Parkinson’s disease (PD), one of the most frequently occurring neurodegenerative diseases in people of middle and older age [2,3], affects the integrity of this network due to degeneration of dopaminergic nigrostriatal projections [4]. Current diagnosis of PD relies on the presence of the typical motor symptoms (hypo- and bradykinesia, rigidity, tremor and impaired balance) and improvement of these symptoms by dopaminergic treatment [5]. Together with validated scales, such as the Hoehn & Yahr (H&Y) Staging and the Unified Parkinson Disease Rating Scale (UPDRS), a global status of the patient’s motor impairment can be presented [6].

However, to date no tests are available to objectively monitor more subtle changes in motor function that may already be present at the most early stages of PD [7]. Visuomotor coordination tasks may provide such a test. So far, visuomotor coordination tests have only been applied in early-stage PD to investigate ocular- or hand motor control separately. Using the oculomotor approach, changes in initiation and execution of saccades have been reported, in particular the inability to suppress reflexive saccades towards extrinsic goals [8-10]. Results from visuomotor studies with a focus on hand coordination, suggest that preparation and execution of goal directed hand movements as well as accuracy of pointing to remembered targets are early signs of sensorimotor degradation in PD [11,12]. These results emphasize the degradation of the basal ganglia at the level of integration of visual input and formation of motor plans in PD [13]. Since eye and hand movements are precisely coordinated in time and position, the combination of both may provide a better model to study visuomotor integration [14]. Eye and hand movements share the internal representation of the goal and both require nigro-striatal connections. However, the internal transformations and effector commands for eyes and hands are quite different [15]. As a result, eye movements may lead or lag the hand movement depending on the task [16]. The relative timing parameters may already be disturbed at an early stage of PD and provide a behavioral marker for early diagnosis of PD. The main goal of this study is to quantify visuomotor coordination in early-stage PD patients using eye-hand coordination tasks with varying complexity.

## Materials and methods

### Participants

Eye and hand movements were recorded in 15 patients with early-stage PD and 15 age-matched controls (> 45 years). Patients were recruited from the neurology outpatient clinic of the Erasmus Medical Center Rotterdam (EMCR), The Netherlands. PD was diagnosed by a neurologist according to the UK Parkinson's Disease Society Brain Bank Diagnostic Criteria for Parkinson’s Disease [17]. Frontal executive function was determined with the frontal assessment battery (FAB) [18]. The Mini-Mental State Examination (MMSE) was assessed to confirm normal cognitive function of all participants [19]. In addition, PD patients had to meet a score of ≤ 2.5 on the H&Y scale as a measure for early-stage PD [20]. The motor section of the UPDRS was examined in PD patients to test motor condition at the moment of participation [21]. Dyskinesia, coexistence of other neurological or psychiatric disorders and ocular pathology were exclusion criteria. For PD patients, dopaminergic treatment was permitted Written consent was obtained from all participants. Prior to the measurements, participants were informed and familiarized with the experimental procedure and equipment. The study was approved by the Medical Ethical Committee of the EMCR.

## Materials and methods

### Experimental procedures

Participants performed four eye-hand coordination tasks in a fixed order: a pro-tapping task, a dual planning task, an anti-tapping task and a spatial memory task. Each task consisted of eight trials, preceded by a maximum of three practice trials. Task instructions were only given before the practice trials.

#### Measurement setup

The measurement setup consisted of a touch screen (ELO Touchsystems), an eye tracking system (Chronos Vision, Berlin, Germany) and an infrared hand-motion capture system (Vicon, Oxford, UK) (see Additional file 1: Figure S1) synchronized by a trigger signal. Participants sat with the head on a chin rest 460 mm in front of the touch screen (viewing angle 75° × 46° (width × height)). Eye movements were sampled at 200 Hz. Prior to the measurement, calibration targets were shown at 20° up, down, left and right from the central position. Coordinates and timing parameters of finger pointings from the touch screen were sampled at 60 Hz (delay 5 ms). Each finger touch or release was registered with custom Matlab programs (MathWorks, Natick, MA, USA). Kinematics of the hand was recorded with the Vicon (200Hz, delay 30 ms). Reconstructed 3D coordinates of each marker were stored online.

**Figure 1:**
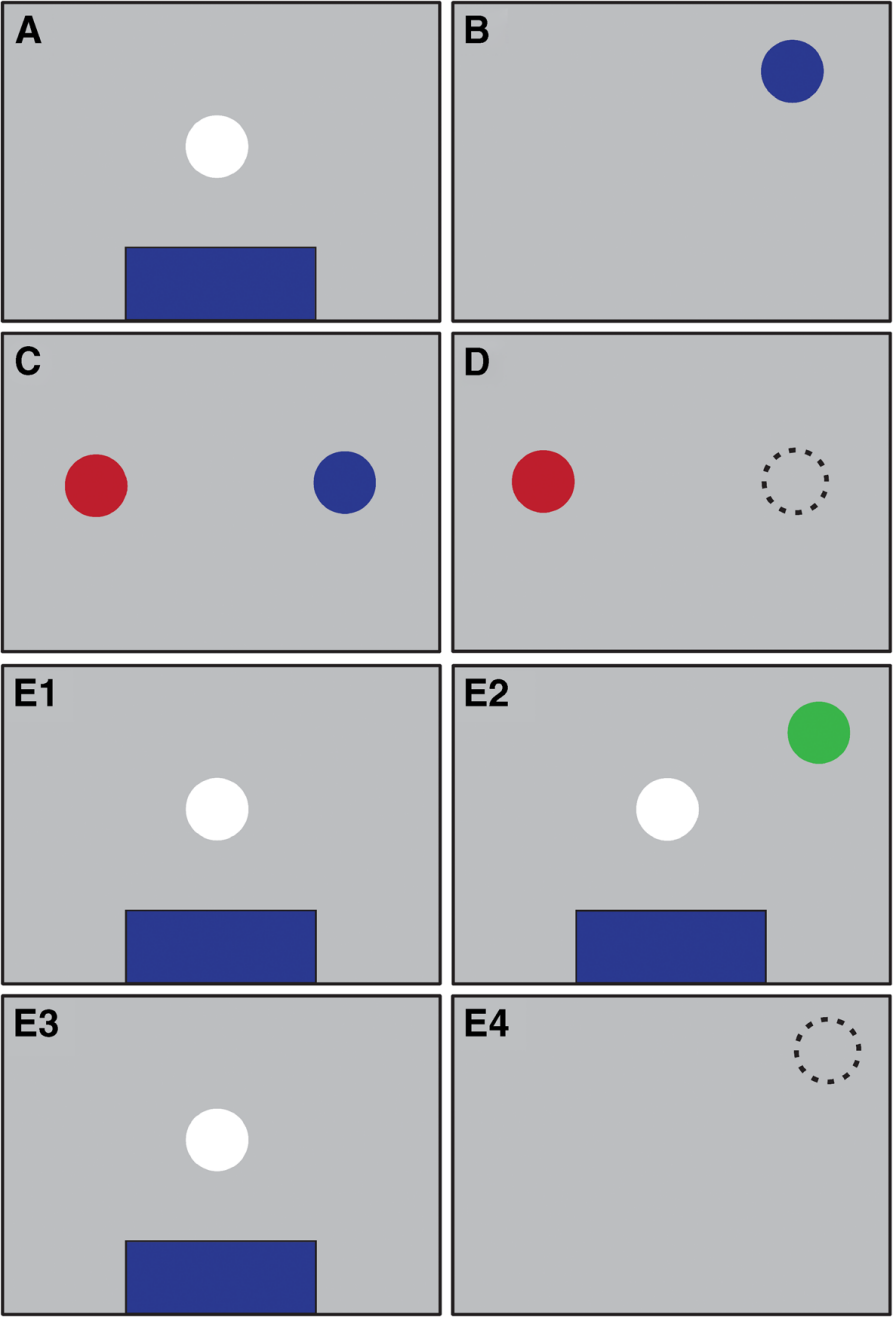
**Overview of the touch screen representations of the starting position and each eye-hand coordination task. A**: starting position. The background color of the touch screen was set to grey (RGB value [0.6 0.6 0.6]) and remained this color during all tasks. The participant fixated the eyes on the white central dot and placed the index finger on the blue bar on the bottom of the screen. **B**: pro-tapping task. A blue dot appeared that had to be touched as fast and accurate as possible. **C**: in the dual planning task, the eyes had to be fixated on the red dot while simultaneously the blue dot had to be touched. **D**: the anti-tapping task required participants to touch the screen on the side opposite of the location of the red dot, mirrored in the y-axis. **E**: In the spatial memory task, a green dot briefly flashed on the screen while participants were in starting position (E1-3). The instruction was to touch the remembered location of the flashed dot as soon as the starting position disappeared.

At the start of each task, participants had to fixate for 2 seconds a white centered target dot and place their index finger on a blue bar at the bottom of the screen, followed by presentation of a target (size 2° visual angle) randomly positioned between 4 and 20° viewing angle (Figure 1).

#### Task description

##### Pro-tapping task

Participants were instructed to touch a blue dot that appeared on the screen as fast and accurate as possible.

##### Dual planning task

A blue and a red dot appeared at opposite positions (mirrored in x-axis and in y-axis) on the touch screen. Participants were instructed to direct gaze towards the red dot while simultaneously the blue dot had to be touched as accurately as possible within 10 seconds. Subjects were instructed not to make eye movements towards the blue dot.

##### Anti-tapping task

A red dot appeared on either the left or right half of the touch screen. Position of the dot varied along the x-axis. Subjects were instructed not to make an eye and hand movement towards the dot, but to touch its virtual location at the opposite side of the screen. After 4 seconds, a control dot appeared as a feedback signal at the correct location during 2 seconds.

##### Spatial memory task

While participants were in starting position, a green dot was flashed for 50 ms. Participants were instructed to touch the remembered location of the flash as accurately as possible within 4 seconds as soon as the starting position disappeared. Subjects were not allowed to make an eye or hand movement before the starting position had disappeared. After 4 seconds, a control dot was presented at the target location for 2 seconds.

### Data analysis and statistics

**Figure 2:**
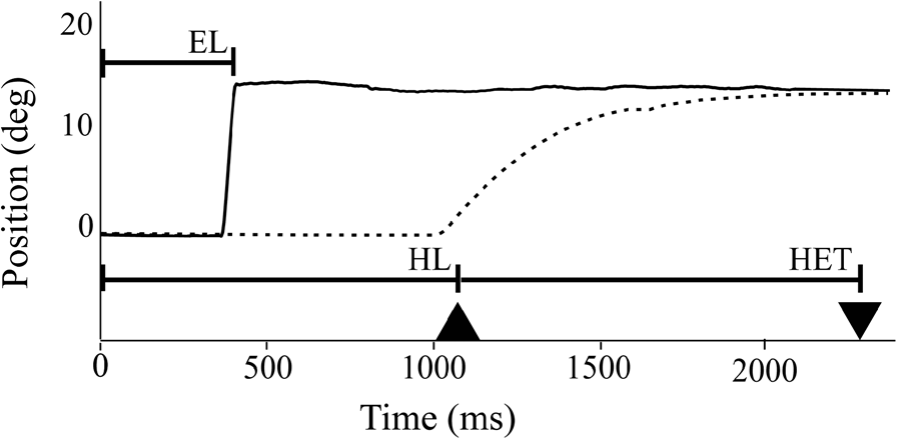
**Quantification of timing variables from eye and hand movement signals.** Traces of eye (solid line) and hand movements (dashed line) during the performance of a pro-tapping task trial. At time 0 ms, the blue dot which is the target location for both eye and hand movement, is displayed at a position of +15 degrees with respect to the central dot. The triangle (▲) represents the moment the finger is released from the touch screen, whereas the reversed triangle (▼) represents the moment the target dot is touched. Eye latency (EL) was defined as the time between presentation of the target and the start of the saccade towards it. Hand latency (HL) is the time between presentation of the target and the release of the finger from the screen (▲). Hand execution time (HET) is the time between the release of the finger from the screen and the touch of the target (▲ to ▼). The kinematic variable hand maximal velocity (HMV) is determined from the peak velocity of the hand between start and end of a hand movement.

Eye and hand movement traces were visually checked and analyzed using custom Matlab programs (Mathworks, Natick, MA, USA). Trials were excluded when there were no sufficient eye or hand movement data (invalid data due to e.g. pupil detection errors or hand marker detection errors). First, the general task performance for each eye-hand coordination task was analyzed. Those trials that were not performed according to the task instructions were classified as incorrect. For each incorrect trial the type of error was listed. For analysis of timing and kinematic variables, a participant was included when at least three of eight trials were performed correctly. Correctly performed trials were quantified according to the following timing and kinematic variables (Figure 2):

**Eye latency** (EL): time between presentation of the target and the start of the saccade (velocity threshold 50 degrees/s) towards the target.

**Hand latency** (HL): the time between presentation of the target and the start of the hand movement towards the target, defined as the moment the finger was released from the blue bar of the touch screen.

**Hand execution time** (HET): the time between the start of the hand movement and the moment the target was touched.

**Hand maximal velocity** (HMV): the peak velocity between start and end of the hand movement.

Variables falling outside the range of 2 SD of the group mean value were identified and rechecked in the analysis software. In total, less than 1% of data was classified as outlier. PD patients and controls were compared with respect to MMSE score, FAB score, age and gender. The means for age, MMSE and FAB score were examined by Student’s *t* test, and Pearson Chi-Square (*χ*2) analyses were used to evaluate the comparability of the groups for gender. For each task, all variables tested had normal distributions (one-sample Kolmogorov-Smirnov test). During the performance of each eye-hand coordination task, group differences in the timing and kinematic variables were analyzed with the multivariate analysis of variance (one-way MANOVA). EL, HL, HMV and HET were the dependent variables and the participant diagnostic status (PD patients and age-matched controls) was the independent variable. In addition, correlations between motor UPDRS scores and the variables EL, HL, HMV and HET were tested for significance using the Pearson’s correlation test. Significance levels were set at P < 0.05.

## Results

15 PD patients (10 men, 5 women, mean age 61.1 ± 8.4 years (mean ± SD)) and 15 age-matched controls (6 men, 9 women, mean age 56.0 ± 6.4 years) were successfully included in our study. Table 1 specifies general characteristics of the participants such as gender, MMSE and FAB scores, and specific characteristics of PD patients such as motor UPDRS and H&Y scores, drug use, and estimated disease duration. No significant between group differences were found for gender (Pearson *χ*^2^(1) = 2.143, p = 0.143), age (t(26.2) = 1.890, p = 0.070), MMSE score (t(24.2) = −1.437, p = 0.163) and FAB score (t(27.8) = 0.841, p = 0.407). One participant was excluded from analysis in two tasks due to pupil detection failure. The number of participants that were not able to perform a task in accordance with the instructions provided is specified below. For each task, the visual targets were presented at a random position on the screen. Eye and hand latency were not significantly correlated with the location of the visual target (EL: p = 0.638, HL: p = 0.970; Pearson’s correlation test), thus eccentricity of a target did not affect eye and hand latencies. One-way MANOVA for eye-hand coordinated performance revealed a significant multivariate main effect for participant diagnostic status in the pro-tapping task (F(4,218) = 11.092, p < 0.001; Wilk’s λ = 0.831), dual planning task (F(4,80) = 8.954, p < 0.001; Wilk’s λ = 0.691), anti-tapping task (F(4,129) = 11.425, p < 0.001; Wilk’s λ = 0.738) and spatial memory task (F(4,138) = 7.422, p < 0.001; Wilk’s λ = 0.823). For each task, the results in terms of performance as well as timing and kinematic variables are presented below.

**Table 1:** Characteristics of the study population (n = 30)

	**PD patients (n = 15)**		**controls (n = 15)**	
**Characteristics**	**Mean ± SD**	**Range**	**Mean ± SD**	**Range**
Age, years*	61.1 ± 8.4	44 - 73	56.0 ± 6.4	49 - 70
Number of women**	5 (33.3%)	-	9 (60%)	-
MMSE score, points*	29.3 ± 0.9	27 - 30	29.7 ± 0.6	28 - 30
FAB score, points*	16.5 ± 1.4	14 - 18	16.9 ± 1.2	15 - 18
Motor section of UPDRS, points	8.9 ± 4.7	4 - 19	-	-
H&Y, points	1.1 ± 0.3	1 - 2	-	-
Duration of disease, years	3.7 ± 2.4	0.66 - 9	-	-

*Age: p = 0.070; MMSE score: p = 0.163; FAB score: p = 0.407 (Student’s *t* test).

**Gender: p = 0.715 (Pearson *χ*2 analyses).

SD = Standard Deviation.

MMSE = Mini-Mental State Examination.

FAB = Frontal Assessment Battery.

UPDRS = Unified Parkinson’s Disease Rating Scale.

H&Y = Hoehn and Yahr staging.

### Pro-tapping task

**Figure 3:**
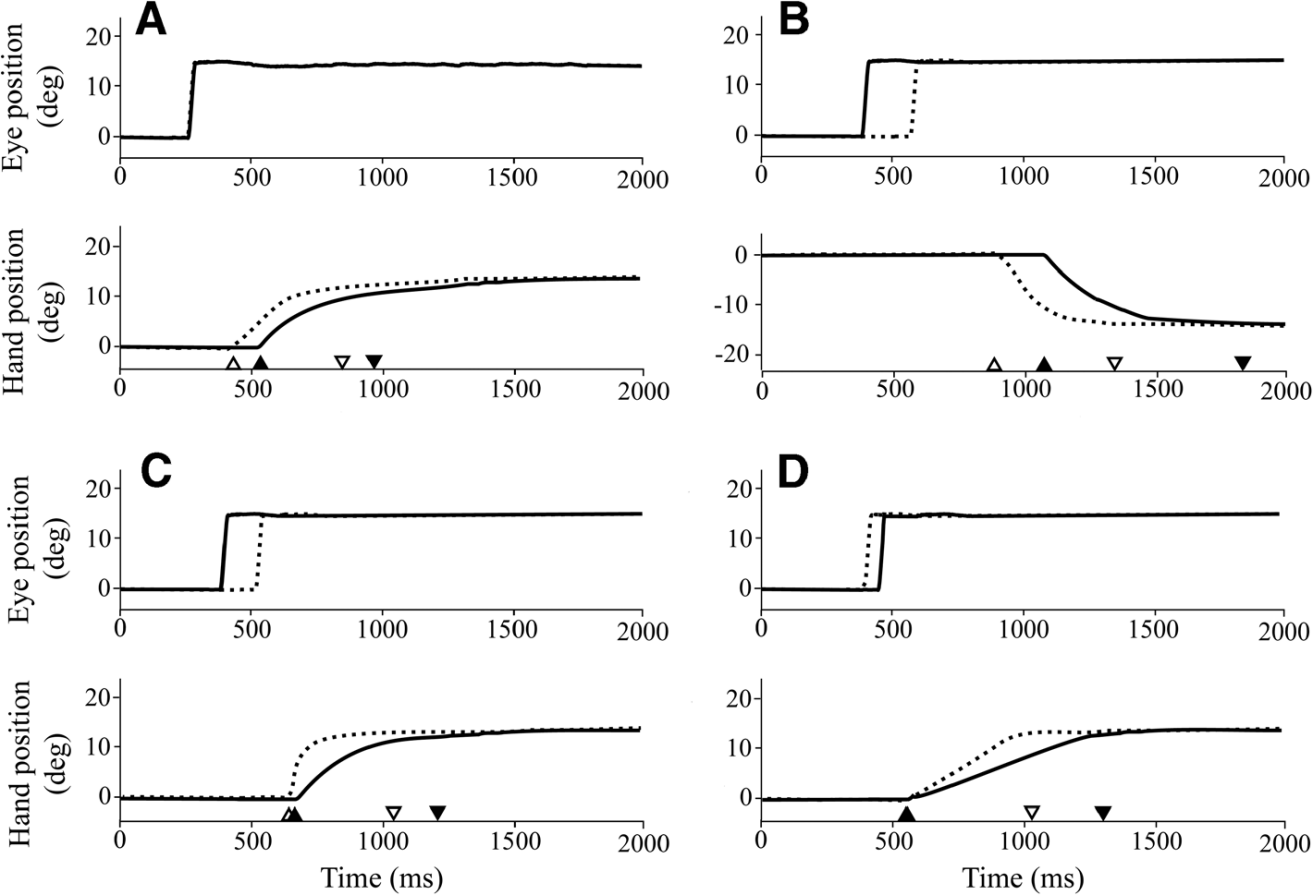
**Representative eye and hand movement traces performed during the four different tasks.** Each panel labeled A, B, C and D consists of two subpanels. The upper subpanel shows the eye movements, the lower subpanel those of the hand. Solid lines: PD patient data, dashed line: control subject data. **Panel A: pro-tapping task.** At time 0 ms the target for the eyes is displayed at a position of 15 degrees from the central dot. Note that in this situation PD patients are able to initiate a saccade towards the target as fast as controls and that PD patients initiated the hand movement significantly slower (HL), as the release of the finger from the screen (▲) was delayed compared to controls (Δ). This panel also shows that the time between the release of the finger from the screen and the touch of the target was significantly increased in PD patients (▲ to ▼) compared to controls (Δ to ∇). **Panel B: dual planning task.** PD patients initiated the saccade towards the target dot significantly faster compared to controls. Note that for the PD patient the release of the finger from the screen (▲) was significantly delayed compared to controls (Δ). The example also shows that the time between the release of the finger from the screen and the touch of the target was significantly increased in PD patients (▲ to ▼) compared to controls (Δ to ∇). **Panel C: anti-tapping task.** The PD patient was significantly faster to initiate an eye movement towards the opposite direction of the displayed dot than the control. The PD patient was able to initiate the hand movement (▲) as fast as controls (Δ). The example also shows that HET was significantly increased in PD patients (▲ to ▼) compared to controls (Δ to ∇). **Panel D: spatial memory task.** The PD patient initiated a saccade towards the remembered target location about as fast as the control. PD patients were also able to initiate the hand movement (▲) as fast as controls (Δ). The time between the release of the finger from the screen and the touch of the target was significantly increased in PD patients (▲ to ▼) compared to controls (Δ to ∇).

All 30 participants performed the pro-tapping task correctly. Figure 3 panel A, shows representative eye and hand movements during the pro-tapping task for a control and PD patient. Initiation of reflexive eye movements (pro-saccades) was not altered in PD patients compared to controls, as EL was not significantly different between groups (F(1,223) = 0.037, p = 0.849; univariate test). However, both the initiation and execution of the hand movement were significantly slower in PD patients (HL: F(1,223) = 21.759, p < 0.0125; HET: F(1,223) = 7.624, p < 0.0125; HMV: F(1,223) = 15.163, p < 0.0125). See Table 2 for timing and kinematic variables for PD patients and controls.

**Table 2:** Between subject effects for participants diagnostic status as independent variable of the four eye-hand coordination tasks using univariate analysis

	**PD Patients**	**Controls**		
**Pro-tapping task**	**mean ± SD**	**mean ± SD**	F-value*	p-value*
Eye Latency (ms)	255 ± 70	255 ± 70	0.037	0.849
Hand Latency (ms)	510 ± 90	455 ± 85	21.759	0.000
Hand Max Velocity (mm/s)	575 ± 180	680 ± 215	15.163	0.000
Hand Execution Time (ms)	480 ± 165	420 ± 130	7.624	0.006
Dual planning task	mean ± SD	mean ± SD	F-value*	p-value*
Eye Latency (ms)	430 ± 165	555 ± 245	7.072	0.009
Hand Latency (ms)	1080 ± 420	860 ± 250	8.767	0.004
Hand Max Velocity (mm/s)	470 ± 185	575 ± 190	6.674	0.012
Hand Execution Time (ms)	745 ± 350	490 ± 180	18.496	0.000
Anti-tapping task	mean ± SD	mean ± SD	F-value*	p-value*
Eye Latency (ms)	430 ± 145	515 ± 165	8.264	0.005
Hand Latency (ms)	670 ± 175	640 ± 125	1.488	0.225
Hand Max Velocity (mm/s)	490 ± 90	580 ± 125	20.352	0.000
Hand Execution Time (ms)	535 ± 170	430 ± 145	14.477	0.000
Spatial memory task	mean ± SD	mean ± SD	F-value*	p-value*
Eye Latency (ms)	475 ± 165	435 ± 115	3.170	0.077
Hand Latency (ms)	570 ± 100	585 ± 105	0.427	0.514
Hand Max Velocity (mm/s)	540 ± 180	670 ± 210	14.429	0.000
Hand Execution Time (ms)	735 ± 310	580 ± 175	15.002	0.000

SD = Standard Deviation.

* = univariate ANOVA.

### Dual planning task

Figure 3, panel B, shows representative eye and hand movement traces of a PD patient and a control during the dual planning task. This task was difficult for most participants. In 56% of the trials PD patients made an error, compared to 44% in controls. The vast majority of errors was caused by an erroneous eye movement towards the hand target. One PD patient was excluded from analysis. In total 8 PD patients and 10 controls were included for further analysis. Initiation of an eye movement towards the target was significantly faster in PD patients than in controls (EL: F(1,85) = 7.072; p < 0.0125), while initiation and execution of the hand movement were significantly slower in PD patients compared to controls (HL: F(1,85) = 8.767, p = 0.004; HET: F(1,85) = 18.496, p < 0.0125; HMV: F(1,85) = 6.674, p < 0.0125) (Table 2).

### Anti-tapping task

Figure 3, panel C, shows representative eye and hand movement traces of a PD patient and a control during the anti-tapping task. One PD patient was excluded from analysis. Percentage of errors due to failure to suppress an eye movement towards the target was 36% in PD patients and 11% in controls. In 11% of PD patients and 14% of controls no eye movement was made at all. Although these trials were not scored as incorrect, they were excluded from further analysis. Overall, timing and kinematic variables were analyzed of 10 PD patients and 14 controls. PD patients initiated the eye movement significantly faster (EL: F(1,134) = 8.264, p < 0.0125) and executed the hand movement significantly slower than controls (HET: F(1,134) = 14.477, p < 0.0125; HMV: F(1,134) = 20.352, p < 0.0125) (see Table 2).

### Spatial memory task

Figure 3, panel D shows representative eye and hand movements during the spatial memory task for both a PD patient and a control. 2 PD patients and 1 control were excluded from further analysis. Overall, the total number of incorrect trials was higher in PD patients (31%), compared to controls (17%). These errors were caused by the inability to suppress an eye and/or hand movement towards the flashed target. PD patients did not make an eye movement at all in 5% of the trials, compared to 12% in controls. These trials were excluded from analysis. Statistical analysis (Table 2) revealed that only execution of the hand movement was significantly slower in PD patients compared to controls (HET: F(1,143) = 15.002, p < 0.0125; HMV: F(1,143) = 14.429, p < 0.0125).

### Correlation of timing variables with motor UPDRS

In the pro-tapping and dual planning task motor UPDRS scores were only significantly correlated with latency of the hand movement (HL: p < 0.001; Pearson correlation test). However, in the anti-tapping task, motor UPDRS score was only correlated with the initiation of the eye movement (EL: p < 0.05). The variables measured in the spatial memory task were not significantly correlated with motor UPDRS scores.

## Discussion

In this study we show that timing and kinematics of eye hand coordination undergo task specific changes in early-stage PD patients.

The initiation of reflexive eye movements in a pro-tapping task is not different from controls. In a meta-analysis study it was concluded that reflexive saccades could be initiated either faster or slower in PD patients, depending on e.g. target eccentricity and materials and method used [22]. Our findings agree with the absence of a fixed impairment in latency of reflexive saccades in early-stage PD.

Eye movements in the dual planning and anti-tapping task, where the target is based on an intrinsic goal, are faster initiated in PD patients. This can be interpreted as a difficulty for PD patients to inhibit reflexive saccades towards both intrinsic and extrinsic targets. In contrast, initiation of the hand movement was delayed in the pro-tapping and dual planning task. The increase in hand latency suggests that in early-stage PD transformation of visuo-spatial information into a motor plan is delayed for the hand, when there is either an intrinsic or extrinsic goal to reach for. Execution of hand movements was slower in every eye-hand coordination task, which is one of the general signs of PD.

A reduced eye latency and prolonged hand latency in the dual planning task could have been a strategy of PD patients to execute the task in a stepwise way. However, this explanation is unlikely in view of reduced eye movement latency of PD patients in reflexive saccade tasks [9,23-26]. In a recent study on perceptual discrimination, PD patients showed hyper-reflexive saccade initiation even though PD patients made more errors compared to controls. In this study it was suggested that top-down attention processes may cause abnormal saccade facilitation, where as a simple pro-saccade task does not show differences in saccade initiation between PD patients and controls [27].

In the anti-tapping task, execution of hand movements was significantly slower in PD patients compared to controls. Correct performance of this task towards an intrinsic goal requires internally triggered eye movements. PD patients initiated these eye movements faster than controls. The decrease in eye latency in our PD patients is in contrast with the prolonged saccadic latencies described in other anti-saccade studies [8,9]. However, in those studies the patients were in a more advanced disease stage of PD.

The faster initiation of eye movements in PD patients compared to controls in the dual planning and anti-tapping task is paralleled by an increase in performance errors. This is in agreement with difficulty in multi-tasking in PD patients [28]. Errors were always due to failure of saccade suppression towards an extrinsic target. PD patients have a reduced blood flow in the dorsolateral prefrontal cortex (DLPFC) [29]. As the DLPFC normally inhibits unwanted reflexive saccades [30], our findings suggest that voluntary inhibitory control over the reflexive saccade system is reduced at an early stage of PD [9].

In the spatial memory task, PD patients made relatively more errors than controls due to problems with the inhibition of eye and/or hand movements towards the target location. Analysis of timing variables of the spatial memory task showed no significant differences in either eye or hand latency between PD patients and controls. This may be due to the 2 s delay before eye and hand movements are initiated during which participants have time to already prepare their actions in this task. Because a longer delay time improves accuracy of the eye movement in a memory-guided saccade task in PD patients [31], one aim for future studies could be to investigate to what extent a variable duration of the delay influences accuracy, timing and kinematic variables.

An important issue of this study is that all PD patients were on dopaminergic treatment. Dopaminergic drug use was also permitted in the study of Chan and colleagues [9]. In a study on the effect of dopaminergic drugs on saccades in PD, Levodopa prolonged reaction time of reflexive saccades and improved the accuracy of voluntary saccades [32]. Considering that dopaminergic treatment may prolong the latency of reflexive eye movements, it is possible that without pharmacological treatment our PD patients would have shorter eye latencies in the pro-tapping task. We found a correlation between motor UPDRS score and hand latency in the pro-tapping and dual planning task, whereas the motor UPDRS score was correlated with eye latency in the anti-tapping task. The UPDRS is commonly used to score e.g. motor symptoms. However, assessment is subjective and also susceptible to placebo effects [7]. Therefore, assessing eye-hand coordination may be useful for other medical studies to objectively score motor symptoms. It may even be an interesting behavioral marker for early-stage diagnosis of PD.

This study shows that relative and absolute timing of eye and hand movements are changed in early-stage PD in a task specific manner. We are planning a larger study to investigate eye-hand coordination in PD patients with different H&Y disease stages. We expect that this study will reveal variable timing aspects of hand coordination in relation to progression of the disease. Furthermore it will be of particular interest to test whether the faster initiation of saccades towards intrinsic goals in eye-hand coordination tasks changes in advanced disease stages.

## Competing interest

This study was funded by Erasmus MC, department of Neuroscience. None of the authors mentioned above report a conflict of interest.

## Authors’ contribution

**DM** Research project: organization and execution, Statistical analysis: Execution, Manuscript: Writing of the first draft Stock Ownership in medically-related fields none, Consultancies none, Advisory Boards none, Partnerships none, Honoraria none, Grants none, Intellectual Property Rights none, Expert Testimony none, Employment Erasmus MC, research assistant, Contracts none, Royalties none, Other none. **SV** Research project: organization and execution Statistical analysis: Execution Manuscript: Writing of the first draft Stock Ownership in medically-related fields none, Consultancies none, Advisory Boards none, Partnerships none, Honoraria none, Grants none, Intellectual Property Rights none, Expert Testimony none, Employment Erasmus MC, research assistant, Contracts none, Royalties none, Other none. **JJMP** Research project: organization and execution, Statistical analysis: Design, Manuscript: Review and critique Stock Ownership in medically-related fields none, Consultancies none, Advisory Boards none, Partnerships none, Honoraria none, Grants none, Intellectual Property Rights, Expert Testimony none, Employment Erasmus MC, staff, Contracts none, Royalties none, Other none. **AJWB** Research project: conceptional ideas and selection of patient population, clinical organization, Statistical analysis: review, Manuscript: Review and critique Stock Ownership in medically-related fields none, Consultancies none, Advisory Boards Royal Visio, Partnerships none, Honoraria none, Grants, Intellectual Property Rights none, Expert Testimony none, Employment Erasmus MC, neurologist, Contracts none, Royalties none, Other none. **JS** Research project: Conception, organization, Statistical analysis: review. Manuscript: Review and critique. Stock Ownership in medically-related fields none, Consultancies none, Advisory Boards none, Partnerships none, Honoraria none. Intellectual Property Rights, Expert Testimony none, Employment Erasmus MC, neuroscientist, group leader, director, Contracts none, Royalties none, Other none. All authors read and approved the final manuscript.

## Supplementary Material

Additional file 1 **Figure S1.** Photograph of measurement setup. The setup consisted of a touch screen, a Vicon motion capture system and a Chronos eye tracker system. Participants were seated in front of the touch screen (A), on which the tasks were displayed. Cameras of the Vicon motion capturing system (B1) registered movements of three reflective markers (B2) attached to a wristband that the participants wore during task performance. The Chronos eye tracker (C) was used to record eye movements during the tasks. Click here for file
