# Supplementary figures and images for: Changes in Timing and kinematics of goal directed eye-hand movements in early-stage Parkinson’s disease
Source: Transl Neurodegener. 2013 Jan 9;2:1. doi: 10.1186/2047-9158-2-1 (PMC3563471; doi:10.1186/2047-9158-2-1)

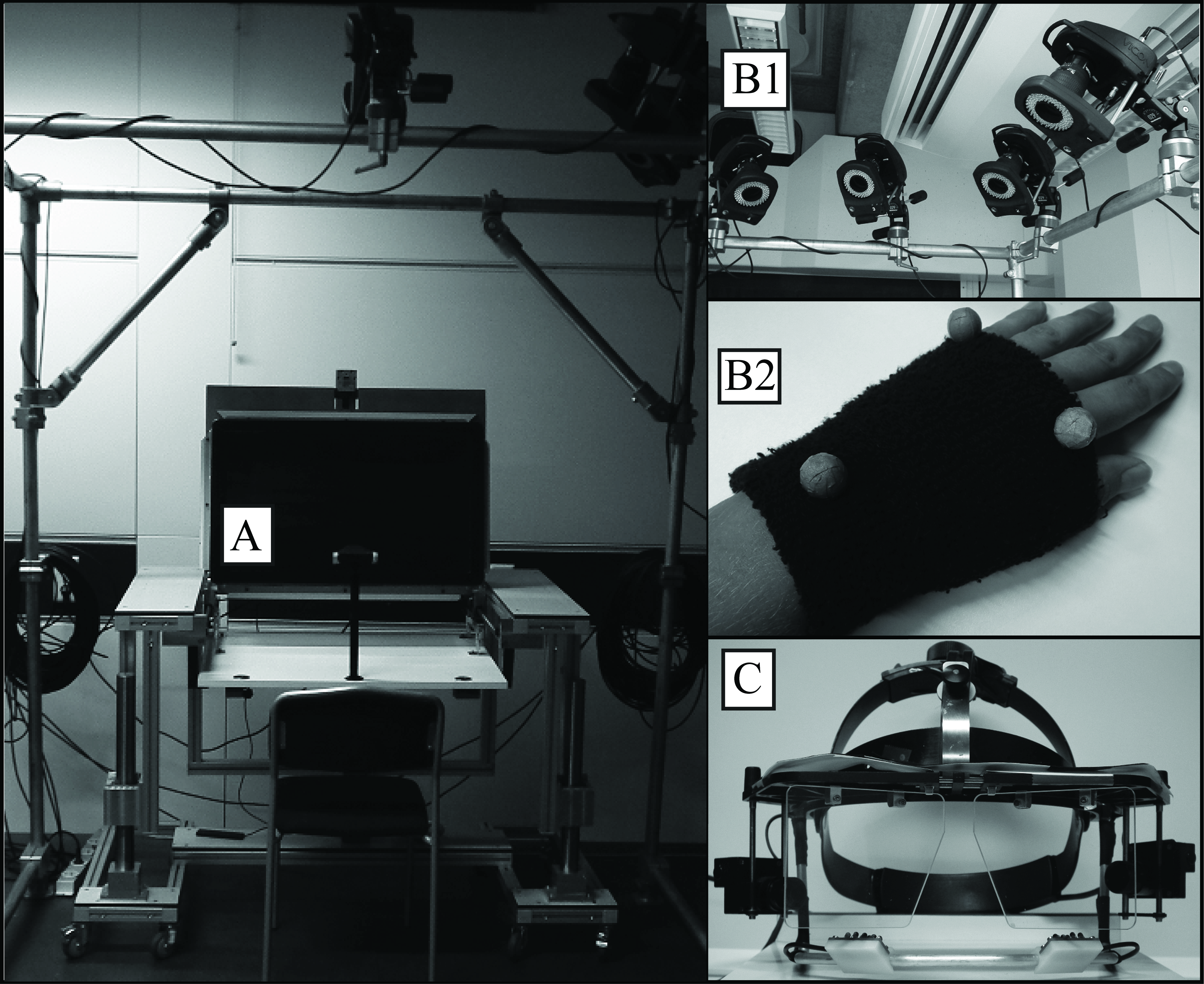

Supplement: Additional file 1 — Figure S1. Photograph of measurement setup. The setup consisted of a touch screen, a Vicon motion capture system and a Chronos eye tracker system. Participants were seated in front of the touch screen (A), on which the tasks were displayed. Cameras of the Vicon motion capturing system (B1) registered movements of three reflective markers (B2) attached to a wristband that the participants wore during task performance. The Chronos eye tracker (C) was used to record eye movements during the tasks. [file 2047-9158-2-1-S1.tiff]
